# Potentially functional polymorphisms in the *ERCC2* gene and risk of Esophageal Squamous Cell Carcinoma in Chinese populations

**DOI:** 10.1038/srep06281

**Published:** 2014-09-11

**Authors:** Mei-Ling Zhu, Jing He, MengYun Wang, Meng-Hong Sun, Li Jin, Xiaofeng Wang, Ya-Jun Yang, Jiu-Cun Wang, Leizhen Zheng, Jia-Qing Xiang, Qing-Yi Wei

**Affiliations:** 1 Cancer Institute, Fudan University Shanghai Cancer Center, Fudan University, Shanghai, China; 2 Department of Oncology, Shanghai Medical College, Fudan University, Shanghai, China; 3 Department of Oncology, Xin Hua Hospital Affiliated To Shanghai Jiao Tong University School of Medicine, Shanghai, China; 4 Department of Pathology, Fudan University Shanghai Cancer Center, Fudan University, Shanghai, China; 5 State Key Laboratory of Genetic Engineering and MOE Key Laboratory of Contemporary Anthropology, School of Life Sciences and Institutes of Biomedical Sciences, Fudan University, Shanghai, China; 6 Fudan-Taizhou Institute of Health Sciences, 1 Yaocheng Road, Taizhou, Jiangsu, China; 7 Department of Thoracic Surgery, Fudan University Shanghai Cancer Center, Fudan University, Shanghai, China; 8 Duke Cancer Institute, Duke University Medical Center, 10 Bryn Searle Dr., Durham, NC 27710, USA

## Abstract

*ERCC2* is indispensable for nucleotide excision repair pathway, and its functional polymorphisms may be associated with cancer risk. In a large case-control study of 1126 esophageal squamous cell carcinomas (ESCC) patients and 1131 controls, we genotyped two SNPs in *ERCC2* (rs238406 G > T and rs13181 T > G) and assessed their associations with ESCC risk. We found a significantly elevated ESCC risk associated with the rs238406 T variant genotypes (adjusted OR = 1.30 and 1.24, 95% CI = 1.02–1.66 and 1.03–1.49 for TG and TG/TT, respectively, compared with GG), particularly in the subgroup of those smoked more than 16 pack-years. Multivariate logistic regression analysis suggested a possible multiplicative gene-environment interaction between rs238406 genotypes and smoking (*P*_interaction_ = 0.026) on ESCC risk. Although no significant risk associations were observed for rs13181, further mini meta-analysis with our and 18 other published studies of 5,012 cases and 8,238 controls found evidence of an association between the rs13181 variant G allele and esophageal cancer risk (TG/GG vs. TT, OR = 1.17; 95% CI = 1.02–1.33). Interestingly, we consistently found a significant correlation between variant genotypes of these two SNPs and *ERCC2* mRNA expression. These findings suggest that potentially functional SNPs in *ERCC2* may contribute to ESCC risk.

China is one of the countries with the highest incidence and mortality of esophageal cancer in the world. Esophageal squamous cell carcinomas (ESCC) account for 90% of all the cases in China^1^. Epidemiological studies have revealed that tobacco smoking, alcohol intake, nutritional deficiencies and dietary carcinogen exposure may contribute to the etiology of ESCC^1^, but only a small proportion of exposed individuals actually develop esophageal cancer, suggesting that genetic factors may also play a vital role in susceptibility to ESCC.

Internal and external environmental exposures, including physical, chemical, and biological carcinogens, can damage cellular DNA, resulting in changes of DNA structure and sequences, thus increasing genomic instability of proliferating cells^2^. At least four main, partly overlapping DNA repair pathways exist in humans for repairing DNA damage^2^. In mammals, nucleotide excision repair (NER) is the most versatile DNA repair mechanism responsible for removing a wide variety of helix-distorting lesions that intervene in base pairing and generally destruct transcription and normal replication^2^. Therefore, reduced DNA repair capacity (DRC) may confer susceptibility to cancer. It is widely recognized that the variation of individual DRC is determined by genetic factors, such as functional single nucleotide polymorphisms (SNPs) of the core genes in DNA repair pathways, which may be the molecular mechanisms underlying the inter-individual variation of DRC in the general population.

Excision repair cross complementing group 2 (*ERCC2*, also known as xeroderma pigmentosum complementation D, *XPD*), is one of the core genes involved in transcription-coupled NER^2^. The protein encoded by this gene has evolutionarily conserved ATP-dependent DNA helicase activity, responsible for unwinding DNA around the lesion site, a crucial step to initiate the NER process^3^. The ERCC2 protein also participates in DNA transcription as an integral member of the basal transcription factor BTF2/TFIIH complex^4^. The importance of ERCC2 is highlighted by the existence of three different disorders that are caused by hereditary defects in this protein, including cancer-prone syndrome xeroderma pigmentosum, Cockayne syndrome, and trichothiodystrophy^4^.

Since the association of the *ERCC2* Lys751Gln (rs13181) polymorphism with ESCC risk was first reported in 2002^5^, there are additional investigations of the association between Lys751Gln and risk of ESCC among different ethnicities^6^,^7^,^8^, but the results have been mixed or conflicting, likely due to a relatively small sample size in each of the published studies. Interestingly, published genome-wide association studies (GWASs) of ESCC in Chinese populations did not identify rs13181 SNP as a susceptible locus^9^,^10^,^11^,^12^, perhaps owing to the stringent *P* values required to avoid false-positive findings, which dramatically decrease the possibility to reveal the modest effect of some common SNPs on risk of cancer, particularly for those SNPs that are potentially functional. Therefore, in the present study, we further investigated the association of two potentially functional *ERCC2* SNPs, including rs13181, with ESCC risk in a large study of an Eastern Chinese population. In addition, we also explored the molecular mechanisms underlying the positive associations.

## Results

### Population characteristics

Population characteristics were described previously^13^. In brief, the cases and controls were adequately matched by age and sex. However, there were a higher proportion of smokers, drinkers and BMI < 25.0 in the cases than in the controls, which were further adjusted for in later multivariate logistic regression analyses (Supplemental Table 1).

### Association between *ERCC2* SNPs and ESCC risk

All the observed genotype frequencies for *ERCC2* SNPs agreed with the Hardy-Weinberg equilibrium in the controls (*P* > 0.05). In the single-locus analyses, we found a significantly elevated ESCC risk associated with the rs238406 T variant genotypes (adjusted odds ratio (OR) = 1.30 and 1.24, 95% confidence interval (CI) = 1.02–1.66 and 1.03–1.49 for TG and TG/TT, respectively, compared with GG). However, these significant risk associations were not observed for the rs13181 SNP (Table 1).

In the combined analysis, we categorized all putative risk (OR > 1.0) genotypes from each SNP into a new variable according to the number of risk genotypes (i.e., *ERCC2* rs238406 TG/TT + rs13181 TG/GG). As a result, we found that individuals carrying “≥1” risk unfavorable genotypes exhibited an increased ESCC risk (adjusted OR = 1.26, 95% CI = 1.03–1.54), compared with those carrying “0” unfavorable genotypes. Such a cumulative effect was dose-dependent, because the risk of ESCC significantly increased with an increasing number of the observed risk genotypes (adjusted OR = 1.23, 95% CI = 1.00–1.50 for one risk genotype; adjusted OR = 1.56, 95% CI = 1.10–2.21 for two risk genotypes; *P*_trend_ = 0.007) (Table 1).

In the stratified analysis, we found that those who carried the rs238406 variant TG/TT genotypes had a significant increased risk, particularly in males (adjusted OR = 1.26, 95% CI = 1.02–1.55), subjects with the cumulative smoking dose >16 pack-years (adjusted OR = 1.58, 95% CI = 1.17–2.13) and subjects with BMI ≥ 25.0 (adjusted OR = 1.40, 95% CI = 1.06–1.84). We also found that the increased risk was more evident in older subjects who carried the rs13181 TG/GG variant genotypes (adjusted OR = 1.44, 95% CI = 1.03–2.01). Likewise, we found similar results for those carrying “≥1” risk genotypes among subgroups of older subjects, males, never-drinkers and subjects with the cumulative smoking dose >16 pack-years. However, further homogeneity tests suggested that there were no differences among all strata, except for subjects with the cumulative smoking dose >16 pack-years by the rs238406 TG/TT genotypes (*P* for homogeneity = 0.036) (Table 2), suggesting a possible interaction. Indeed, we did find a statistical evidence for a multiplicative gene-environment interaction between rs238406 genotypes and smoking (*P*_interaction_ = 0.026) on ESCC risk.

We finally calculated the false-positive report probability (FPRP) values for all observed significant findings. With the assumption of a prior probability of 0.1, the FPRP values were 0.136, 0.192, 0.091, 0.096 and 0.094, respectively, for associations of rs238406 TG/TT genotypes, 1 combined risk genotype, 2 combined risk genotypes and “≥1” combined risk genotypes with an increased risk of ESCC in all subjects, as well as in the subgroups of the cumulative smoking dose >16 pack-years. The FPRP values were all <0.20, suggesting that these significant associations were noteworthy (Table 3).

### Meta-analysis of *ERCC2* SNPs with ESCC risk

**Figure 1:**
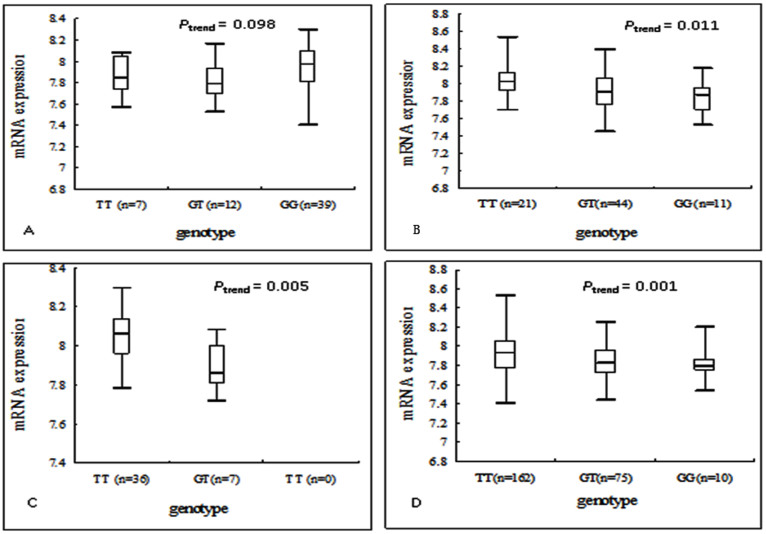
Effect of two SNPs on ERCC2 mRNA expression for different populations in EBV-transformed lymphoblastoid cell lines. (A) the effect of rs238406 on mRNA expression for 58 Asians; (B) the effect of rs238406 on mRNA expression for 76 Europeans; (C) the effect of rs13181 on mRNA expression for 43 Chinese Han, Beijing (CHB); (D) the effect of rs13181 on mRNA expression for 247 subjects with different ethnicities.

In addition to the present association study, we identified 18 other published case-control studies on *ERCC2* rs13181 SNP^6^,^7^,^8^,^14^,^15^,^16^,^17^, with the sample sizes ranging between 151 and 1600, for which we performed a mini meta-analysis to assess the association of this SNP with ESCC risk (Supplemental Table 2). We found that the rs13181 variant G allele was significantly associated with an increased risk of esophageal cancer (OR = 1.17; 95% CI = 1.02–1.33 for TG/GG vs. TT) based on 5,012 cases and 8,238 controls in the pooled analysis as well as in subgroup analysis of these ESCC studies (OR = 1.19; 95% CI = 1.01–1.40 for TG/GG vs. TT) (Table 4, Figure 1). The leave-one-out sensitivity analysis validated the stability of the results (data not shown). The shapes of the funnel plots seemed symmetrical, and Egger's test further showed no publication bias (Table 4). However, we were not able to do a similar mini meta-analysis for rs238406, because there was only one published case-control study on esophageal adenocarcinoma cancer in a European population^18^.

### Correlation analysis for *ERCC2* mRNA expression levels and variant genotypes

**Figure 2:**
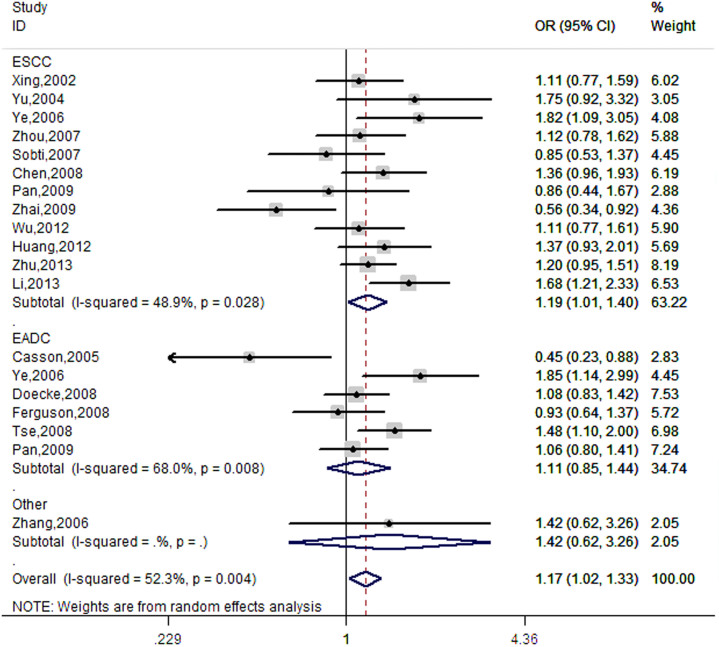
Forest plot of overall esophageal cancer risk associated with ERCC2 rs13181 polymorphism (TG/GG vs. TT) by the random-effects for each of the 19 studies. For each study, the estimates of OR and its 95% CI were plotted with a box and a horizontal line. The symbol filled diamond indicates pooled OR and its 95% CI.

To explore possible underlying molecular mechanisms, we performed the *ERCC2* genotype-phenotype correlation analysis using the available data on the genotypes and mRNA expression levels of lymphoblastoid cell lines derived from peripheral lymphocytes from 270 people (ref. 43). As a result, we found a statistical significance for rs13181 allele T → G on gene transcription expression (*P*_trend_ = 0.005 and 0.001 for CHB and all populations, respectively) (Figure 2), which supports the finding of an association between this SNP and ESCC risk. For rs238406, however, we found a statistically significant trend for the allele G → T effect on *ERCC2* mRNA expression in Europeans (*P*_trend_ = 0.011) but a borderline significance in CHB (*P*_trend_ = 0.098).

## Discussion

In the present study, we found that two potentially functional *ERCC2* SNPs (i.e. rs238406 G > T and rs13181 T > G) were individually or collectively associated with ESCC risk. We also observed a multiplicative gene-environment interaction between rs238406 genotypes and smoking on ESCC risk. Because many studies have investigated the associations between *ERCC2* rs13181 T > G SNP and risk of ESCC cancer, we also performed a mini meta-analysis that provided additional statistical evidence of such an association.

The *ERCC2* gene, mapped to chromosome 19q13.3, comprises 23 exons and encodes 760 amino acids. Acting as a single-strand DNA-dependent ATPase, and also a 5′-3′ DNA helicase, the ERCC2 protein participates in both DNA unwinding during NER and transcription initiation by binding to the transcription factor IIH via p44^19^,^20^. Mutations in *ERCC2* could result in transcription defects and abnormal apoptosis by reducing the BTF2/TFIIH activity, thus leading to a severe but variable depression of NER^21^. ERCC2 also has a second function that is involved in base excision repair (BER) of oxidative base damage of the transcribed strand of transcriptionally active genes^21^. It has been demonstrated that an increased risk for ESCC in Chinese populations was associated with reduced mRNA expression of *ERCC2* as detected in peripheral blood mononuclear cells^22^. *ERCC2* mRNA expression levels may also be of importance, as it has been shown that mRNA levels of *ERCC2* are correlated with the DNA repair capacity phenotype in primary lymphoblasts^23^.

Given the role of the *ERCC2* gene in the DNA repair pathway, it is believed that some subtle alterations in *ERCC2* gene functions are more readily tolerated. Individuals with inherited *ERCC2* defects display their disease phenotype in a recessive genetic model, because only the homozygotes are prone to accumulate genetic damage and may have a marked predisposition to cancer. However, the present study found some dominant effects of *ERCC2* SNPs on ESCC risk, perhaps owing to complex gene-gene or gene-environment interactions that may have some impact on genetic models for disease risk^24^. This hypothesis needs to be tested in additional larger studies.

Previous studies found that *ERCC2* rs238406 conferred susceptibility to cancers of the bladder^25^, lung^26^ and other organs. Only one reported study focused on esophageal cancer and found no association between rs238406 and esophageal adenocarcinoma risk with only 56 European patients and 95 healthy controls^18^. In contrast, the present study included over 1000 cases and 1000 controls with a much improved study power and thus found a statistical evidence for a weak association between the rs238406 variant genotypes and increased ESCC risk. SNP rs238406, a silent polymorphism in codon 156 of exon 6, does not change the amino acid residue (Arg156Arg). It is therefore unlikely that the enzymatic function of ERCC2 is affected by this SNP. However, it is likely that these SNPs may influence ERCC2 protein levels through an effect on mRNA splicing as predicted by the SNPinfo software. Such a possible molecular mechanism is biologically plausible. For example, a silent A → T substitution at codon 399 (Val399 Val) of the *phenylalanine hydroxylase* (*PAH*) gene is the major determinant for exon 11 skipping during the pre-mRNA processing step that thereby results in a phenylketonuria (PKU) phenotype^27^. Indeed, we found a trend for the rs238406 allele G → T effect on *ERCC2* mRNA expression for different populations, indicating that it may be a potentially functional SNP. Alternatively, the SNP may be in LD with other untyped functional polymorphisms or with an adjacent susceptibility gene. For example, we found two SNPs (rs3810366 and rs2097215) located in 5′ near gene are in high LD (r2 > 0.8) with rs238406. Furthermore, the region of chromosome 19q13.3, comprising the *ERCC2* gene, encodes several other DNA repair genes, such as *ERCC1*, *XRCC1* and *LIG*^28^. Therefore, additional mechanistic studies are needed to unravel the molecular mechanisms underlying this observed association.

Some meta-analyses have reported that individuals carrying the SNP rs13181 (Lys751Gln) G variant allele had an increased risk of cancers of the lung^29^, stomach^30^, skin^31^ and esophagus^14^,^32^. Our meta-analysis not only confirmed the positive association with risk of esophageal cancer but also provided additional statistical evidence that the G variant allele was associated with an increased risk of esophageal cancer in Chinese populations. The rs13181 SNP, i.e., the T → G base substitution in exon 23, causes an amino acid alteration from lysine to glycine at codon 751, which leads to a complete change in electronic configuration of the amino acid and thus reduces DNA repair efficiency^33^. For example, the TT genotype was reported to be associated with a sub-optimal repair of chromatid aberrations induced by X-irradiation in breast cancer^34^. In the present study, we also found a significant trend for the rs13181 allele T → G effect on *ERCC2* transcript expression levels in different ethnic populations, indicating that the *ERCC2* rs13181 SNP may be a underlying genetic determinant of esophageal cancer risk.

The present study also identified that rs238406 SNP had a significant interaction with tobacco consumption in ESCC risk. NER is an indispensable pathway for the repair of bulky and helical distorting DNA adducts generated by tobacco smoke. Therefore, light smokers may have less DNA damage and less risk for developing advanced tumors stage and grade^35^, while in heavy smokers, the genetic effect may be accelerated by accumulated DNA damage induced by tobacco smoking. This hypothesis needs to be tested in larger studies that allow one to perform sufficient stratified analysis and to explore gene-gene and gene-environment interactions.

However, in recently published GWASs for ESCC^9^,^10^,^11^,^12^,^36^, these two *ERCC2* SNPs were not among the reported top-hits, nor any SNPs in other DNA repair genes. There are some explanations for this. First, GWAS is bases on the theory that the common genetic variants are associated with common human diseases^37^. It is clear that most of the functional SNPs are believed not to be common, nor is cancer. Second, GWASs have an inherent “multiple testing problems” that often require the stringent *P* values to avoid false-positive findings. In that case, it would markedly decrease the power to detect the influence of common SNPs that may have modest impact on risk of cancer. Third, the lack of statistical power makes it difficult to interpret negative findings, such as for those DNA repair genes known to be involved in cancer etiology. These underscore the need to continue the search for such missing etiological factors in the GWAS dataset or in the combined analysis of several published GWASs in the future to identify additional causal but rare or functional variants. Forth, most published GWASs do not include comprehensive information about exposures for additional adjustment or stratification, so the actual associations may be either biased or masked. Indeed, Wu et al recently extended their GWAS findings by additional analyses for gene-environment interactions, in which they have discovered some new genetic contribution to ESCC through the analysis of interaction with alcohol consumption by performing a genome-wide gene-environment interaction analysis^9^. Moreover, GWAS is based on common tagging SNPs with the aim of not to capture all the “causative” but the “representative” SNPs. Therefore, the present study is another extension to the investigation of functional SNPs in cancer etiology and to the needs of identifying additional evidence of biological mechanisms underlying the positive association findings.

In conclusion, the present study demonstrated that potentially functional *ERCC2* SNPs may confer susceptibility to ESCC, possibly by the effects on *ERCC2* mRNA expression levels, suggesting an important role of functional *ERCC2* SNPs in the etiology of ESCC in Chinese populations. Nevertheless, some limitations should be addressed. First, because of tissue access constraint, we did not have the opportunity to examine the levels of *ERCC2* mRNA in the target tissue from the study population. It is likely that the exact mechanisms by which the *ERCC2* variant regulates the gene transcription activity *in vivo* are not the same as those observed in cell lines *in vitro*, which warrants additional *in vivo* mechanistic studies. Second, insufficient study power may be another concern in regard to some negative findings, particularly for the analyses of subgroups with small sample sizes, or for SNPs with a rare variant allele, such as the rs13181 G variant allele. For example, we failed to find any statistical evidence of an association between rs13181 and ESCC risk, but we did find such a statistical evidence in a subsequent meta-analysis of a much larger sample size. Third, we only investigated two potentially functional SNPs in the present study due to financial constraints. As we know, cancer is a complex disease for which any single SNP may not be sufficient for the prediction of the overall risk. Future studies should include more genes and more SNPs with more rigorous study designs for mechanistic studies.

## Methods

### Study subjects

The study populations were described in details previously^13^. Briefly, all subjects were genetically unrelated ethnic Han Chinese from Eastern China. The present study included 1126 cases who had newly diagnosed and histopathologically confirmed primary ESCC from Fudan University Shanghai Cancer Center (FUSCC) between March 2009 and September 2011. We also included 1131 cancer-free controls who were frequency matched to the cases by age (±5 years) and sex, and who were randomly selected from those recruited at the same time period in the Taizhou Longitudinal Study (TZL)^38^. The overall response rate was 93% and 90% for cases and controls, respectively. Having signed a written informed consent, each participant provided information about demographics and environmental exposure history, including age, sex, ethnicity, body mass index (BMI), tobacco and alcohol consumption, and donated 10 mL venous blood sample, of which 1 mL was used for genomic DNA extraction. This research protocol was approved by the Institutional Review Board of FUSCC and the experiment on humans was performed in accordance with relevant guidelines and regulations.

### SNP selection and genotyping

We selected two potentially functional SNPs located in the coding region (i.e., rs13181 T > G and rs238406 G > T) for genotyping, because rs13181 is one of the most reported non-synonymous SNPs that result in an amino acid change, while rs238406 is a synonymous SNP that has a putative function of splicing predicted by the SNP function prediction (FuncPred) software (SNPinfo, http://snpinfo.niehs.nih.gov/). In addition, these two SNPs also satisfy the following criteria: (1) minor allele frequency (MAF) ≥ 5% in Chinese Han, Beijing (CHB) descendants; (2) with low linkage disequilibrium (LD) of an r^2^ < 0.8 for each paired SNPs. The LD analysis suggested that these two potentially functional SNPs also captured other 11 untyped SNPs (r^2^ ≥ 0.8) within the *ERCC2* gene (Supplemental Table 3). We extracted genomic DNA from blood samples and performed genotyping by using the Taqman real-time PCR method, as described previously^13^. The successful genotyping rate for both SNPs was greater than 95%.

### Meta-analysis of *ERCC2* SNPs with ESCC risk

To summarize the published data on these two SNPs, we searched two electronic databases (MEDLINE and EMBASE) for all relevant articles. We defined the search terms, inclusion and exclusion criteria as previously reported^39^. The last search update was on May 20, 2013. We chose the fixed-effects (Mantel–Haenszel method) or random-effects model (DerSimonian and Laird method) based on the heterogeneity test^40^,^41^. When *P* value of the heterogeneity test was <0.05, the random-effects model was used, which indicates the existence of heterogeneous effect sizes across all studies; otherwise, the fixed-effects model was more appropriate. We also conducted sensitivity analyses to evaluate the effect of an individual study on the overall risk of cancer and used the funnel plot and the Egger's linear regression test to examine potential influence of publication bias^42^. Because there were few reported studies on the association of rs238406 G > T SNP in the *ERCC2* gene with ESCC risk, we could not perform its meta-analysis.

### Correlation analysis for ERCC2 mRNA expression levels and variant genotypes

To provide possible underlying mechanisms, we analyzed the correlation between *ERCC2* mRNA expression levels and variant genotypes. The genotyping data were from the HapMap Project (http://hapmap.ncbi.nlm.nih.gov/) consisting of 3.96 million SNP genotypes from 270 individuals of four ethnic groups (CEU, 90 Utah residents from northern and western Europe; CHB, 45 unrelated Han Chinese in Beijing; JPT, 45 unrelated Japanese in Tokyo; YRI, 90 Yoruba in Ibadan, Nigeria), and the data on transcript expression levels were from EBV-transformed B lymphoblastoid cell lines from the same 270 individuals (http://app3.titan.uio.no/biotools/tool.php?app=snpexp)^43^.

### Statistical methods

We examined the Hardy-Weinberg equilibrium for genotype distribution in controls by a goodness-of-fit χ^2^ test. We also used the χ^2^ test to assess the differences in demographic variables, risk factors and genotype frequency distributions between the cases and controls. We computed OR and their 95% CI from univariate and multivariate logistic regression models to estimate the associations of *ERCC2* SNPs with ESCC risk, which were also evaluated in subgroup analyses stratified by demographic variables and risk factors. Finally, we performed linear regression model-trend test for the genotype–phenotype association analysis.

For significant findings observed in the present study, we used FPRP to assess false-positive associations. We calculated FPRP with a favorite prior probability of 0.1 to detect an OR of 0.67/1.50 (protective/risk effects) for an association with genotypes under investigation. A FPRP value < 0.20 was considered a noteworthy association^44^. We performed the meta-analysis with the STATA software, version 11.0 (Stata Corporation, College Station, TX), and other statistical analyses with the SAS software (version 9.1; SAS Institute, Cary, NC). All statistical tests were two-sided with a statistical significance level of *P* < 0.05.

## Author Contributions

Conceived and designed the experiments: Q.Y.W. and J.Q.X. Performed the experiments: M.L.Z. and J.H. Analyzed the data: M.L.Z. and M.Y.W. Contributed reagents/materials/analysis tools: L.J., Y.J.Y., J.C.W., M.H.S., X.F.W. and L.Z.Z. Wrote the paper: M.L.Z. and Q.Y.W. All authors reviewed the manuscript.

## Supplementary Material

Supplementary Information

## Figures and Tables

**Table 1:** Genotype frequencies of the *ERCC2* SNPs and their association with risk of ESCC

	Cases a	Controls a		Crude OR	Adjusted OR	
Variants	No. (%)	No. (%)	*P* b	(95% CI)	(95% CI) b	*P* c
*ERCC2* rs238406, HWE = 0. 066^g^						
GG	325 (28.97)	374 (33.66)	0.057^d^	1.00	1.00	
TG	558 (49.73)	515 (46.35)		1.25 (1.03–1.51)	1.22 (1.00–1.48)	0.053
TT	239 (21.30)	222 (19.98)		1.24 (0.98–1.57)	**1.30 (1.02–1.66)**	**0.038**
TG/TT	797 (71.03)	737 (66.34)	0.017^e^	1.24 (1.04–1.49)	**1.24 (1.03–1.49)**	**0.024**
*ERCC2* rs13181, HWE = 0. 413^g^						
TT	937 (83.51)	954 (85.87)	0.300^d^	1.00	1.00	
TG	175 (15.60)	149 (13.41)		1.20 (0.94–1.51)	1.16 (0.42–1.48)	0.229
GG	10 (0.89)	8 (0.72)		1.27 (0.50–3.24)	1.10 (0.42–2.89)	0.844
TG/GG	185 (16.49)	157 (14.13)	0.122^e^	1.20 (0.95–1.51)	1.16 (0.91–1.47)	0.227
No. of at-risk genotypes f						
0	248 (22.10)	297 (26.73)	0.010		1.00	
1	766 (68.27)	734 (66.07)		1.25 (1.03–1.52)	1.23 (1.00–1.50)	0.050
2	108 (9.63)	80 (7.20)		1.62 (1.16–2.26)	**1.56 (1.10–2.21)**	**0.012**
					***P*_trend_ = 0.007**	
Dichotomized groups						
0	248 (22.10)	297 (26.73)	0.011		1.00	
≥1	874 (77.90)	814 (73.27)		1.28 (1.06–1.56)	**1.26 (1.03–1.54)**	**0.024**

^a^The numbers of each single nucleotide polymorphism were less than the total number of subjects because some genotyping data were unavailable.

^b^Two sides Chi-square test for genotype distributions between cases and controls.

^c^Adjusted for age, sex, BMI, smoking status, and drinking status in logistic regression models.

^d^For additive genetic models.

^e^For dominant genetic models.

^f^The risk-genotypes used for the calculation were *ERCC2* rs238406 TG/TT + rs13181 TG/GG

^g^A goodness-of-fit *χ^2^* test for Hardy Weinberg equilibrium (HWE) for genotype distribution in controls.

**Table 2:** Stratification analysis for associations between *ERCC2* SNPs and ESCC risk

	rs238406				rs13181				Combined effect of risk genotypes			
	(cases/controls)				(cases/controls)				(cases/controls)			
Variables	GG	TG/TT	Adjusted OR (95% CI) a	*P* b	TT	TG/GG	Adjusted OR (95% CI) a	*P* b	0	≥1	Adjusted OR (95% CI) a	*P* b
Age, yr (median)												
≤60	158/178	402/380	1.27 (0.97–1.66)	0.573	477/476	83/82	0.89 (0.63–1.26)	0.158	128/141	432/417	1.17 (0.87–1.56)	0.218
>60	167/196	395/357	1.26 (0.97–1.63)		460/478	102/75	1.44 (1.03–2.01)		120/156	442/397	1.39 (1.05–1.85)	
Sex												
Males	259/290	646/571	1.26 (1.02–1.55)	0.708	757/740	148/121	1.16 (0.88–1.52)	0.943	199/231	706/630	1.26 (1.01–1.58)	0.820
Females	66/84	151/166	1.24 (0.81–1.89)		180/214	37/36	1.18 (0.69–2.02)		49/66	168/184	1.31 (0.83–2.08)	
Pack-years												
0	133/155	295/356	0.94 (0.70–1.25)	**0.036**	363/444	65/67	1.20 (0.82–1.76)	0.717	107/126	321/385	0.97 (0.71–1.31)	0.050
≤16 (mean)	37/85	115/156	1.53 (0.95–2.47)		126/210	26/31	1.38 (0.77–2.50)		27/69	125/172	1.60 (0.95–2.70)	
>16 (mean)	155/134	387/225	**1.58 (1.17–2.13)**		448/300	94/59	1.06 (0.73–1.55)		114/102	428/257	1.58 (1.14–2.20)	
Drinking status												
Never	173/245	451/504	1.26 (0.99–1.60)	0.935	525/648	99/101	1.21 (0.89–1.65)	0.797	131/197	493/552	1.33 (1.02–1.72)	0.782
Ever	152/129	346/233	1.25 (0.92–1.69)		412/306	86/56	1.03 (0.70–1.52)		117/100	381/262	1.20 (0.87–1.66)	
BMI												
<25.0	209/155	505/335	1.14 (0.89–1.47)	0.253	591/427	123/63	1.38 (0.99–1.93)	0.165	154/124	560/366	1.25 (0.95–1.64)	0.748
≥25.0	116/219	292/402	1.40 (1.06–1.84)		346/527	62/94	1.01 (0.71–1.43)		94/173	314/448	1.31 (0.98–1.75)	

^a^Obtained in logistic regression models with adjustment for age, sex, BMI, smoking status and drinking status.

^b^*P* for heterogeneity test using the Chi-square-based Q test.

**Table 3:** False-Positive Report Probability Values for associations between ESCC risk and genotypes of *ERCC2* polymorphisms

				Prior probability				
Genotypes	Positive OR (95% CI) a	*P* value b	Statistical Power c	0.2500	0.1000	0.0100	0.0010	0.0001
rs238406								
TT vs. GG	1.24 (0.98–1.57)	0.075	0.988	0.185	0.406	0.883	0.987	0.999
TG/TT vs. GG	1.24 (1.04–1.49)	0.017	0.975	0.050	0.136	0.633	0.946	0.994
Pack-years > 16	1.49 (1.12–1.98)	0.006	0.523	0.033	0.094	0.532	0.920	0.991
Combined risk genotypes								
No. at-risk genotypes d								
1 vs. 0	1.25 (1.03–1.52)	0.026	0.984	0.073	0.192	0.723	0.964	0.996
2 vs. 0	1.62 (1.16–2.26)	0.005	0.452	0.032	0.091	0.523	0.917	0.991
≥1 vs. 0	1.28 (1.06–1.56)	0.011	0.930	0.034	0.096	0.539	0.922	0.992

^a^The crude OR reported in Tables 2 and 3.

^b^The omnibus chi-square test of the genotype frequency distributions reported in Tables 2 and 3.

^c^Statistical power was calculated using the number of observations in the study and the OR and *P* values in Tables 2 and 3.

^d^Combined risk genotypes were to referred to *ERCC2* rs238406 TG/TT + rs13181 TG/GG.

**Table 4:** Meta-analysis of the association between *ERCC2* rs13181 polymorphism and esophageal cancer risk

	No. of studies	GG vs. TT			TG vs. TT			GG vs. TG/TT			TG/GG vs. TT		
Variables		OR (95% CI)^c^	*P_OR_*^a^	*P*_het_^b^	OR (95% CI)^d^	*P_OR_*^a^	*P*_het_^b^	OR (95% CI)^c^	*P_OR_*^a^	*P*_het_^b^	OR (95% CI)^d^	*P_OR_*^a^	*P*_het_^b^
All	19	**1.28 (1.08–1.52)**	**0.005**	**0.075**	**1.15 (1.01–1.30)**	**0.030**	0.025	1.19 (1.01–1.40)	0.034	0.231	**1.17 (1.02–1.33)**	**0.020**	0.004
Ethnicity													
Asian	11	1.36 (0.97–1.91)	0.078	0.165	1.16 (1.00–1.35)	0.050	0.141	1.28 (0.92–1.80)	0.140	0.176	1.18 (1.01–1.39)	0.115	0.052
Non-Asian	8	1.26 (1.03–1.54)	0.025	0.071	1.14 (0.90–1.43)	0.276	0.019	1.16 (0.97–1.40)	0.110	0.331	1.14 (0.90–1.45)	0.267	0.007
Histological type													
Squamous Cell	12	1.33 (0.98–1.79)	0.064	0.152	1.17 (1.01–1.37)	0.039	0.083	1.20 (0.90–1.60)	0.216	0.200	**1.19 (1.01–1.40)**	**0.034**	0.028
Adenocarcinoma	6	1.26 (1.02–1.55)	0.036	0.048	1.09 (0.85–1.41)	0.500	0.023	1.18 (0.97–1.44)	0.095	0.215	1.11 (0.85–1.44)	0.448	0.008
Other	1	3.13 (0.13–77.85)	0.486	0.076	1.33 (0.57–3.08)	0.507	--	3.03 (0.12–75.19)	0.499	--	1.42 (0.62–3.26)	0.404	--
Publication Bias e			0.955			0.414			0.970			0.497	

^a^*P* value of the Z-test for odds ration test.

^b^*P* value of the Q-test for heterogeneity test.

^c^Fixed-effects model.

^d^Random-effects model.

^e^*P* value of Egger's test for publication bias.
