# Supplementary material for: Potentially functional polymorphisms in the ERCC2 gene and risk of Esophageal Squamous Cell Carcinoma in Chinese populations
Source: Sci Rep. 2014 Sep 11;4:6281. doi: 10.1038/srep06281 (PMC4160711; doi:10.1038/srep06281)
Supplement: Supplementary Information [file srep06281-s1.doc]

**Potentially functional polymorphisms in the *ERCC2* gene and risk of Esophageal Squamous Cell Carcinoma in Chinese populations**

Mei-Ling Zhu ^1,2,3^, Jing He ^1,2^, MengYun Wang ^1,2^, Meng-Hong Sun ^4^, Li Jin ^5,6^, Xiaofeng Wang ^5,6^, Ya-Jun Yang ^5,6^, Jiu-Cun Wang ^5,6^, Leizhen Zheng^3^, Jia-Qing Xiang ^2,7,*^, Qing-Yi Wei ^1,8,*^

^1^Cancer Institute, Fudan University Shanghai Cancer Center, Fudan University, Shanghai, China ^2^Department of Oncology, Shanghai Medical College, Fudan University, Shanghai, China ^3^Department of Oncology, Xin Hua Hospital Affiliated To Shanghai Jiao Tong University School of Medicine, Shanghai, China

^4^Department of Pathology, Fudan University Shanghai Cancer Center, Fudan University, Shanghai, China

^5^State Key Laboratory of Genetic Engineering and MOE Key Laboratory of Contemporary Anthropology, School of Life Sciences and Institutes of Biomedical Sciences, Fudan University, Shanghai, China

^6^Fudan-Taizhou Institute of Health Sciences, 1 Yaocheng Road, Taizhou, Jiangsu, China

^7^Department of Thoracic Surgery, Fudan University Shanghai Cancer Center, Fudan University, Shanghai, China

^8^Duke Cancer Institute, Duke University Medical Center, 10 Bryn Searle Dr., Durham, NC 27710, USA

| **Supplemental Table 1.** Distributions of selected variables in ESCC cases and cancer-free controls in an Eastern Chinese population | | | |
| --- | --- | --- | --- |
| Variables | Controls  No. (%) | Cases  No. (%) | *P* ^a^ |
| All subjects | 1131 (100%) | 1126 (100%) |  |
| Age, yr (Mean ±SD)^b^ | 60.77 ± 10.24 | 60.40 ± 8.30 | 0.125 |
| ≤ 50 | 156 (13.79) | 138 (12.26) |  |
| 51- 60 | 405 (35.81) | 422 (37.48) |  |
| 61-70 | 401 (35.46) | 429 (38.10) |  |
| > 70 | 169 (14.94) | 137 (12.17) |  |
| Sex |  |  | 0.078 |
| Males | 879 (77.72) | 909 (80.37) |  |
| Females | 252 (22.28) | 217 (19.27) |  |
| Smoking status |  |  | 0.0003 |
| Yes | 611 (54.02) | 693 (61.55) |  |
| No | 520 (45.98) | 433 (38.45) |  |
| Drinking status |  |  | < .0001 |
| Yes | 369 (32.63) | 501 (44.44) |  |
| No | 762 (67.37) | 625 (55.56) |  |
| Pack-years |  |  | < .0001 |
| 0 | 520 (45.98) | 429 (38.10) |  |
| ≤ 16 (mean) | 245 (21.66) | 152 (13.50) |  |
| > 16 (mean) | 366 (32.36) | 545 (48.40) |  |
| BMI ^c^ |  |  | <.0001 |
| < 25.0 | 497 (43.94) | 717 (63.68) |  |
| ≥ 25.0 | 634 (56.06) | 409 (36.32) |  |

^a^ Two-sided *χ^2^* test for distributions between cases and controls.

^b^ Data are mean ± SD and *P* value from Student’s *t* test.

^c^ BMI, body mass index.

| **Supplemental Table 2.** Characteristics of studies included in the meta-analysis | | | | | | | | |
| --- | --- | --- | --- | --- | --- | --- | --- | --- |
| Study | Country | Ethnicity | Histological type | Genotyping method | Source of controls | Sample sizes (Cases/controls) | HWE (controls) | MAF (controls) |
| Xing, 2002 | China | Asian | ESCC | PCR-RFLP | HB | 433/524 | 0.87 | 0.07 |
| Yu, 2004 | China | Asian | ESCC | PCR-RFLP | HB | 135/152 | 0.11 | 0.07 |
| Casson, 2005 | Canada | European | EADC | PCR-RFLP | HB | 56/95 | 0.93 | 0.4 |
| Zhang, 2006 | China | Asian | UN | PCR-RFLP | HB | 106/106 | 0.57 | 0.05 |
| Ye, 2006 | Sweden | European | EADC | PCR-RFLP | PB | 96/472 | 0.11 | 0.37 |
| Ye, 2006 | Sweden | European | ESCC | PCR-RFLP | PB | 81/472 | 0.11 | 0.37 |
| Zhou, 2007 | China | Asian | ESCC | PCR-RFLP | HB | 327/612 | 0.82 | 0.08 |
| Sobti, 2007 | Indian | Asian | ESCC | PCR-RFLP | UN | 120/160 | 0.64 | 0.37 |
| Doecke, 2008 | Australia | Mixed | EADC | iPLEXTM | PB | 263/1337 | 0.22 | 0.35 |
| Ferguson, 2008 | Ireland | European | EADC | TaqMan | PB | 208/247 | 0.61 | 0.39 |
| Tse, 2008 | America | Mixed | EADC | TaqMan | HB | 312/453 | 0.72 | 0.34 |
| Chen, 2008 | China | Asian | ESCC | PCR-RFLP | HB | 321/392 | 0.88 | 0.22 |
| Pan, 2009 | America | European | EADC | TaqMan | HB | 346/456 | 0.43 | 0.24 |
| Pan, 2009 | America | European | ESCC | TaqMan | HB | 38/456 | 0.43 | 0.24 |
| Zhai, 2009 | China | Asian | ESCC | PCR-RFLP | HB | 200/200 | 0.12 | 0.13 |
| Wu, 2012 | China | Asian | ESCC | PCR-RFLP | HB | 235/235 | 0.5 | 0.15 |
| Huang, 2012 | China | Asian | ESCC | PCR-RFLP | HB | 213/358 | 0.8 | 0.12 |
| Li, 2013 | China | Asian | ESCC | PCR-RFLP | PB | 400/400 | 0.43 | 0.11 |
| Zhu, 2013 | China | Asian | ESCC | TaqMan | HB | 1122/1111 | 0.413 | 0.07 |

HB, Hospital based; PB, Population based; PCR, polymerase chain reaction; RFLP, Restriction fragment length polymorphisms polymerase chain reaction; HWE, Hardy-Weinberg equilibrium; MAF, minor allelic frequency; UN, undetermined; EADC, esophageal adenocarcinoma; ESCC, esophageal squamous cell carcinoma

| **Supplemental Table 3.** SNPs captured by the selected two *ERCC2* SNPs as Predicted by SNPInfo software | | | | | | | | | | | | | |
| --- | --- | --- | --- | --- | --- | --- | --- | --- | --- | --- | --- | --- | --- |
| **rs** | **Chr** | **Allele** | **LDsnp** | **Splicing**  **(site)** | **Splicing**  **(ESE or ESS)** | **miRNA**  **(Sanger)** | **nsSNP** | **Stop Codon** | **Conservation** | **Nearby**  **Gene** | **Allele** | **Asian** | **CHB** |
| **rs13181** | **19** | **rs13181** | **1** | **Y** | **Y** | **Y** | **--** | **--** | **0.585468** | **ERCC2** | **T** | **0.875** | **0.899** |
| rs1799787 | 19 | rs13181 | CHB/1.000 | -- | -- | -- | -- | -- | 0.119958 | ERCC2 | G | 0.920 | 0.899 |
| rs1799793 | 19 | rs13181 | CHB/0.846 | -- | -- | Y | -- | -- | 0.353901 | ERCC2 | C | 0.916 | 0.933 |
| rs11878644 | 19 | rs238406 | CHB/0.828 | -- | -- | -- | -- | -- | 0 | ERCC2||PPP1R13L | C | 0.511 | 0.494 |
| rs1618536 | 19 | rs238406 | CHB/0.927 | -- | -- | -- | -- | -- | 0 | ERCC2 | C | 0.544 | 0.518 |
| rs171140 | 19 | rs238406 | CHB/0.928 | -- | -- | -- | -- | -- | 0 | ERCC2 | A | 0.570 | 0.524 |
| rs1799783 | 19 | rs238406 | CHB/0.860 | -- | -- | -- | -- | -- | 0.134579 | ERCC2 | T | 0.540 | 0.567 |
| rs2097215 | 19 | rs238406 | CHB/0.830 | -- | -- | -- | -- | -- | NA | ERCC2||PPP1R13L | C | -- | 0.500 |
| rs238403 | 19 | rs238406 | CHB/0.850 | -- | -- | -- | -- | -- | 0 | ERCC2 | C | 0.564 | 0.560 |
| rs238404 | 19 | rs238406 | CHB/0.928 | -- | -- | -- | -- | -- | 0 | ERCC2 | T | 0.544 | 0.524 |
| rs238405 | 19 | rs238406 | CHB/0.860 | -- | -- | -- | -- | -- | 0 | ERCC2 | T | 0.535 | 0.567 |
| **rs238406** | **19** | **rs238406** | **1** | **Y** | **Y** | **--** | **--** | **--** | **0.36557** | **ERCC2** | **G** | **0.548** | **0.500** |
| rs238407 | 19 | rs238406 | CHB/0.860 | -- | -- | -- | -- | -- | 0 | ERCC2 | A | 0.535 | 0.567 |
